# Inverted Papilloma of the Lacrimal Sac and Nasolacrimal Duct: A Case Report and Review of the Literature

**DOI:** 10.7759/cureus.6989

**Published:** 2020-02-14

**Authors:** Ya Fang Amanda Cheang, David Loke

**Affiliations:** 1 Otolaryngology, Head and Neck Surgery, National Healthcare Group, Singapore, SGP; 2 Otolaryngology, Head and Neck Surgery, Khoo Teck Puat Hospital, Singapore, SGP

**Keywords:** inverted papilloma, nasolacrimal duct, lacrimal sac, dacryocystorhinostomy

## Abstract

Inverted papillomas of the lacrimal sac and nasolacrimal duct are exceedingly rare. Though histologically benign, these tumors are locally aggressive, have propensity for recurrence and are associated with a chance of malignant transformation. These tumors can present in an innocuous manner, masquerading as more common conditions such as primary acquired nasolacrimal duct obstruction. We present our experience with one such case and a review of the literature to emphasize the importance of pre-operative assessment and intra-operative vigilance, so as to aid in accurate diagnosis and early treatment.

## Introduction

Inverted papillomas are benign tumors that arise most commonly from the nasal cavity and paranasal sinuses. They rarely arise primarily from the lacrimal sac or nasolacrimal duct. Although histologically benign, these tumors can be locally aggressive and have a tendency to recur following surgical excision. Malignant transformation has been associated with these tumors in a small proportion of cases. We present our experience with a case of inverted papilloma arising primarily from the nasolacrimal system and a review of the literature. 

## Case presentation

**Figure 1:**
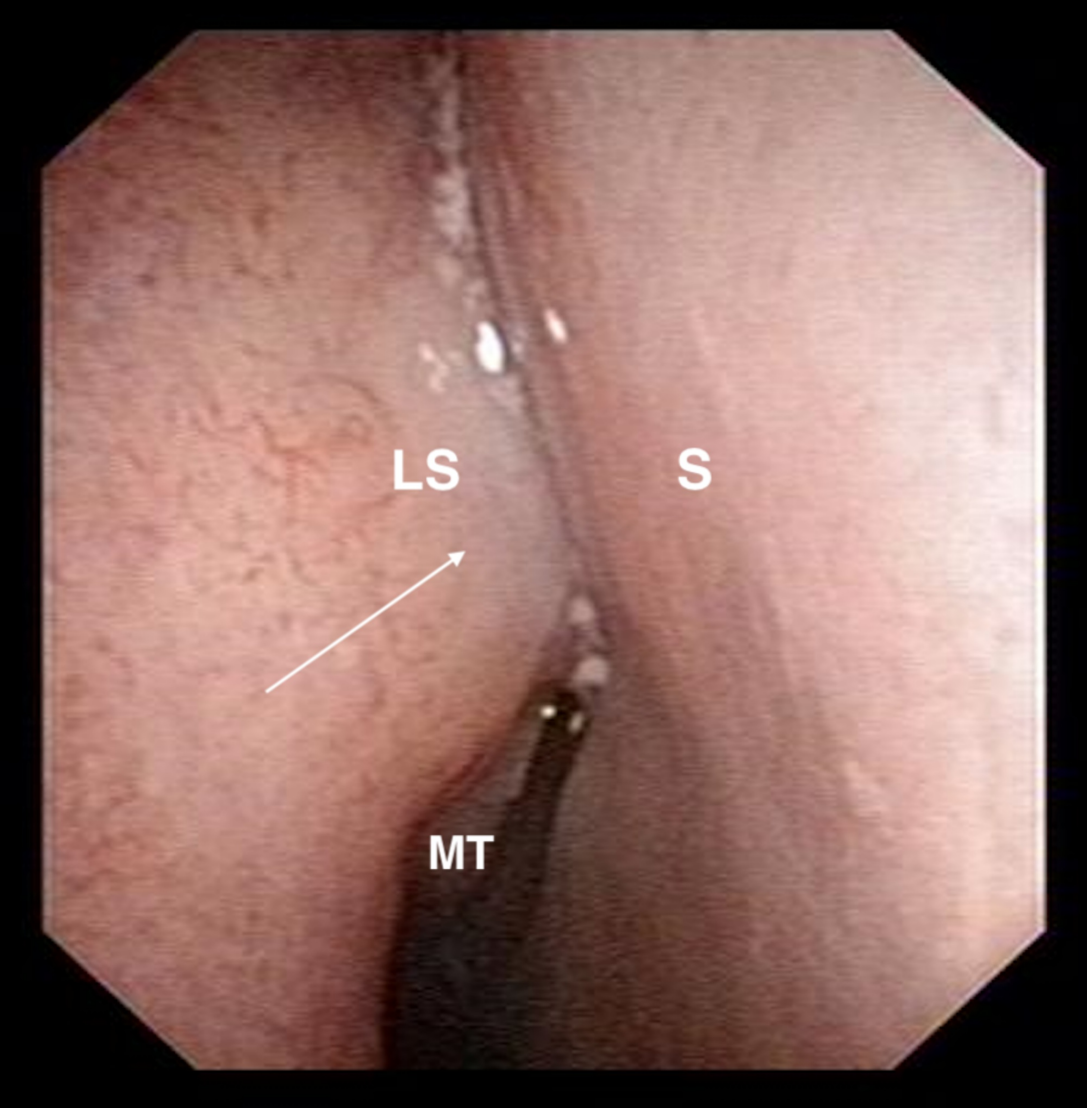
Nasoendoscopy image showing bulge over right lateral nasal wall at approximate site of the lacrimal sac with normal overlying mucosa. LS: approximate site of lacrimal sac; S: septum; MT: middle turbinate. Arrow points to lateral nasal wall bulge.

A 45-year-old male presented with a five-year history of unilateral recurrent epiphora and swelling over the right medial canthal region. On examination, he had mild right hyperglobus, an increased tear lake with delayed dye disappearance test and a hard stop on syringing. Nasoendoscopy showed a bulge over the lateral wall of the nasal cavity, anterior to the middle turbinate (at the approximate site of the lacrimal sac), with normal overlying mucosa (Figure 1).

**Figure 2:**
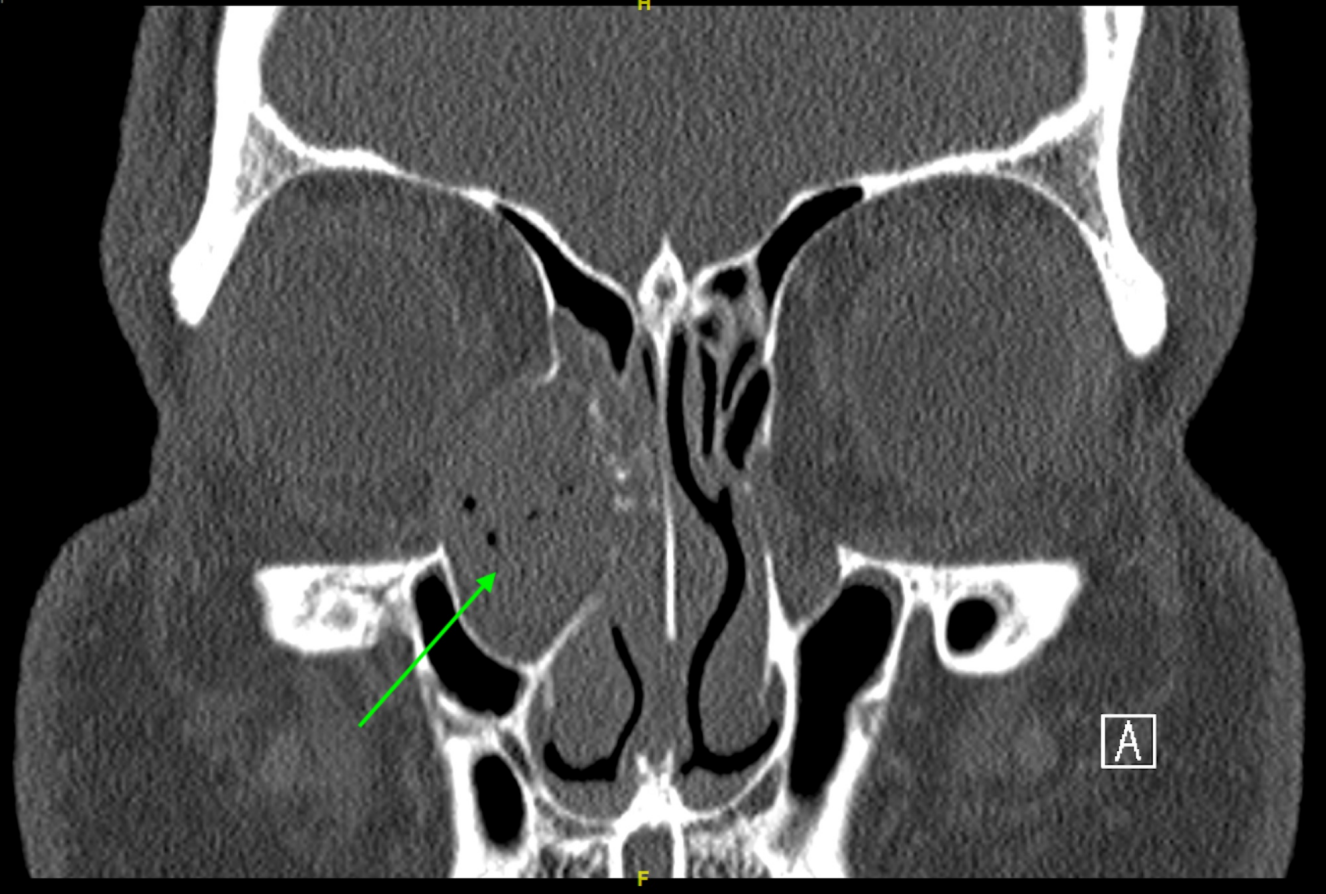
CT scan of orbits showing a 2.6 cm lesion centered upon the right nasolacrimal duct with adjacent bony remodeling and expansion, as well as partial dehiscence of the medial orbital wall.

Computed tomography (CT) scan demonstrated a 2.6 cm lesion centered upon the right nasolacrimal duct with adjacent bony remodeling and expansion, as well as partial dehiscence of the medial orbital wall. Radiological features were deemed to be in keeping with a chronic mucocele (Figure 2).

**Figure 3:**
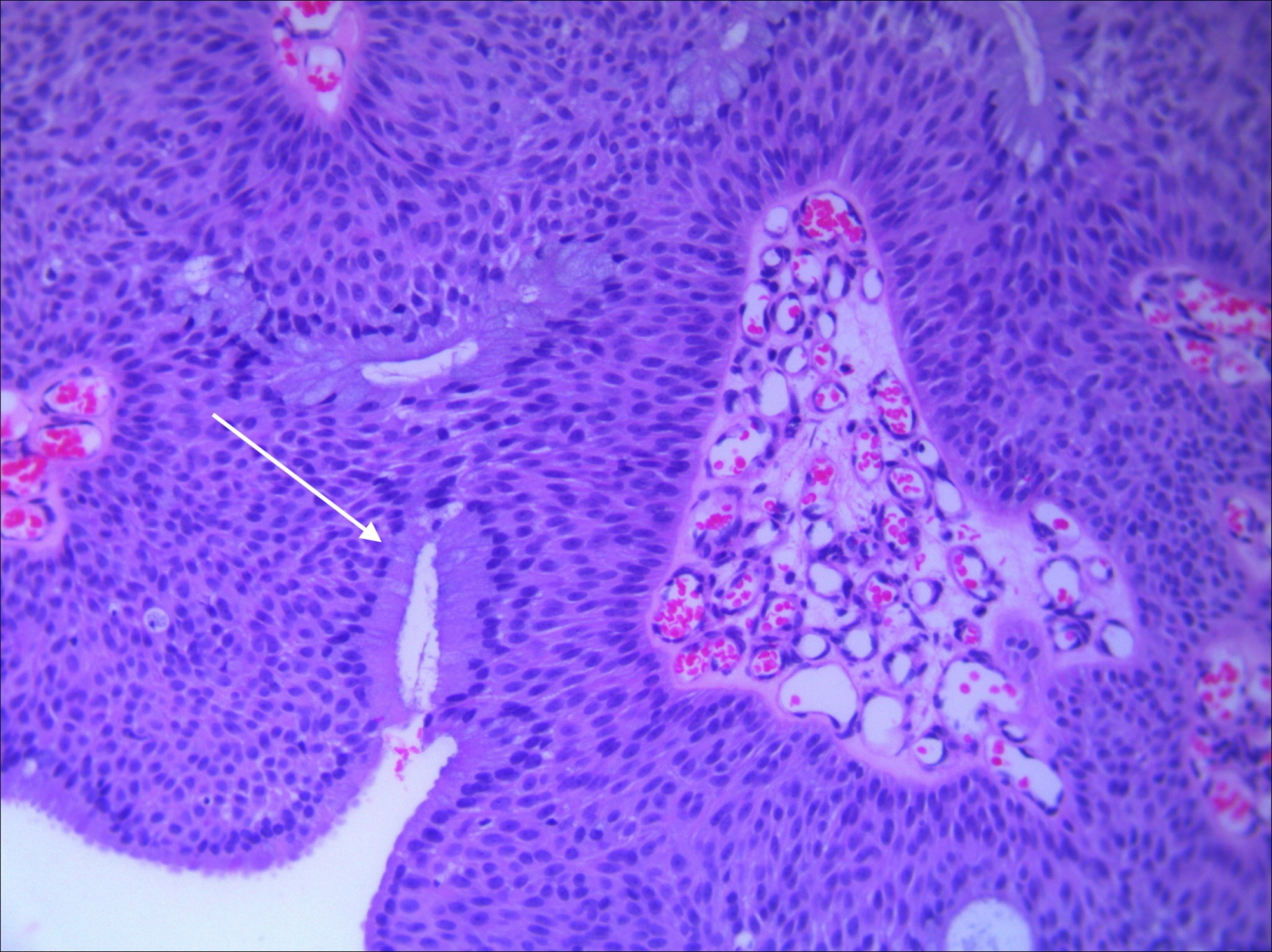
H&E stain of biopsy specimen showing inverted growth pattern.

The patient underwent endoscopic dacryocystorhinostomy. Intra-operatively, a large nasolacimal mucocele with mucopurulent contents was noted. A biopsy of the lacrimal sac mucosa was performed as it appeared abnormally edematous and papillomatous. Histology showed a transitional cell papilloma with both exophytic and inverted growth patterns (Figure 3). No malignant features were noted. Tumor cells stained positively for CK7 and p63. Ki-67 was positive in less than 10% of the tumor cells.

**Figure 4:**
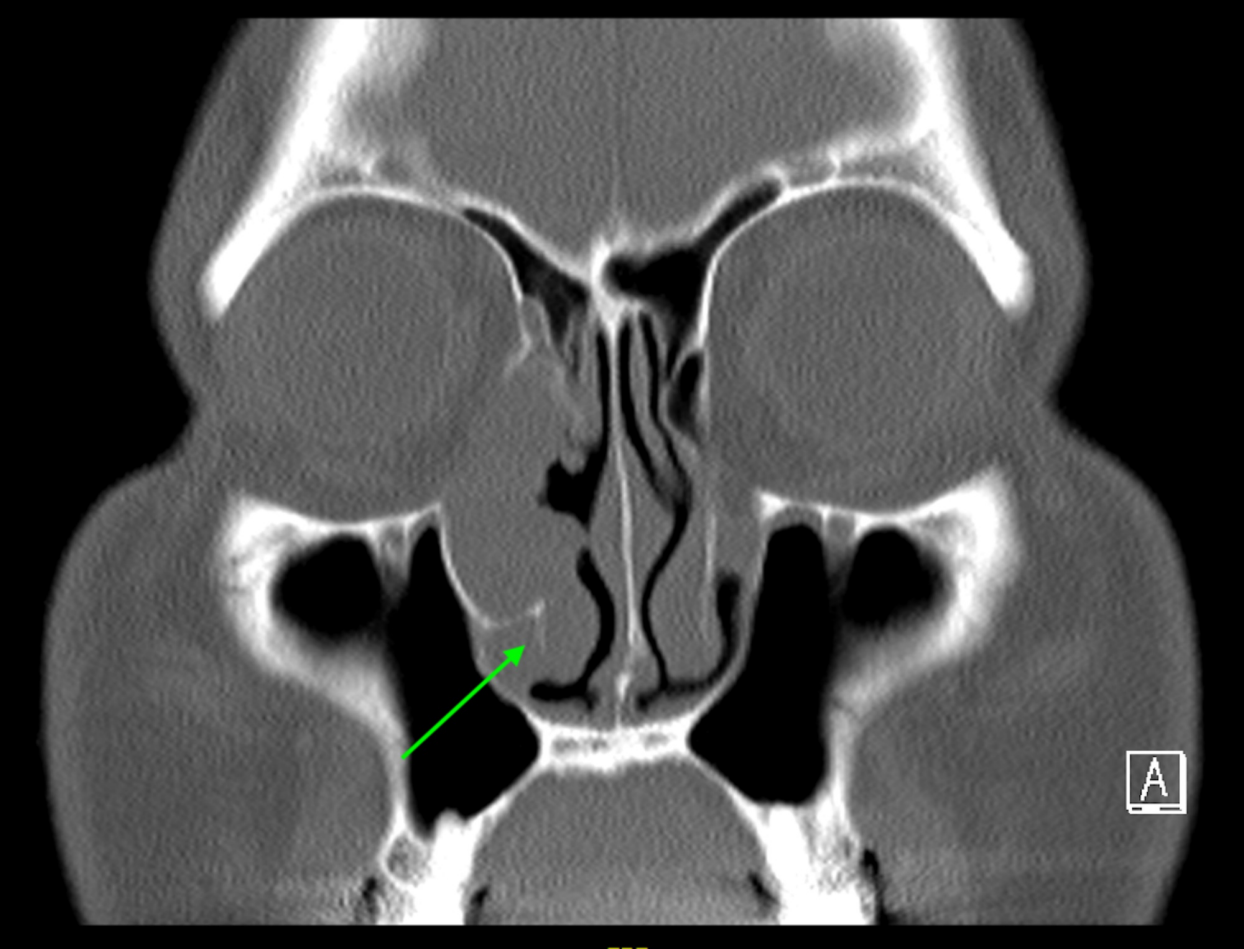
CT sinus done prior to first definitive surgical excision showing extension of mass into the inferior meatus.

The patient was counseled for definitive surgical excision, but he denied surgical intervention for personal reasons. Three months later, a mass was noted at the inferior meatus and a biopsy proved it to be inverted papilloma. A repeat CT scan demonstrated marked bony expansion of the nasolacrimal canal and protrusion of the mass through the inferior meatus, in addition to previous CT findings (Figure 4).

The patient subsequently agreed to surgery and intra-operatively, the tumor was seen to involve the entire nasolacrimal duct, extending beneath the inferior turbinate, with erosion of the uncinate and axilla of the middle turbinate. A combined open and endoscopic resection of the tumor was performed. This included dacryocystectomy and a limited medial maxillectomy via a Lynch incision combined with lateral rhinotomy approach. The medial wall of the maxillary sinus, part of the inferior turbinate and axilla of the middle turbinate were resected. Frozen section and final histology confirmed clear margins.

**Figure 5:**
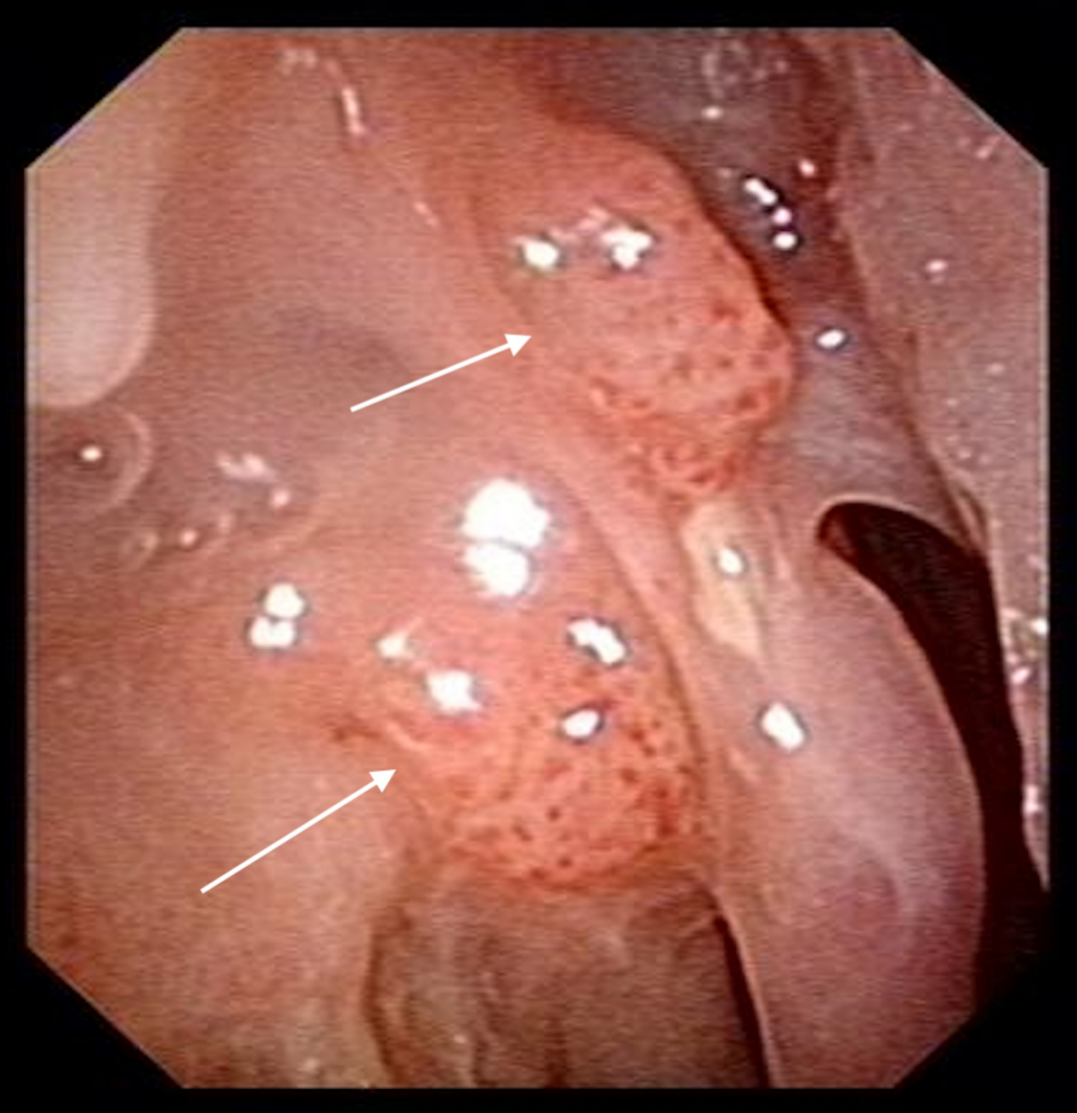
Nasoendoscopy image one year post-operatively showing recurrence of inverted papilloma over the right lateral nasal wall. Arrows point to sites of papilloma recurrence.

The patient was reviewed regularly post-operatively. One year after surgery, he was noted to have recurrence of papillomas over the right lateral nasal wall (Figure 5). A biopsy of these lesions confirmed the diagnosis of recurrent inverted papilloma.

**Figure 6:**
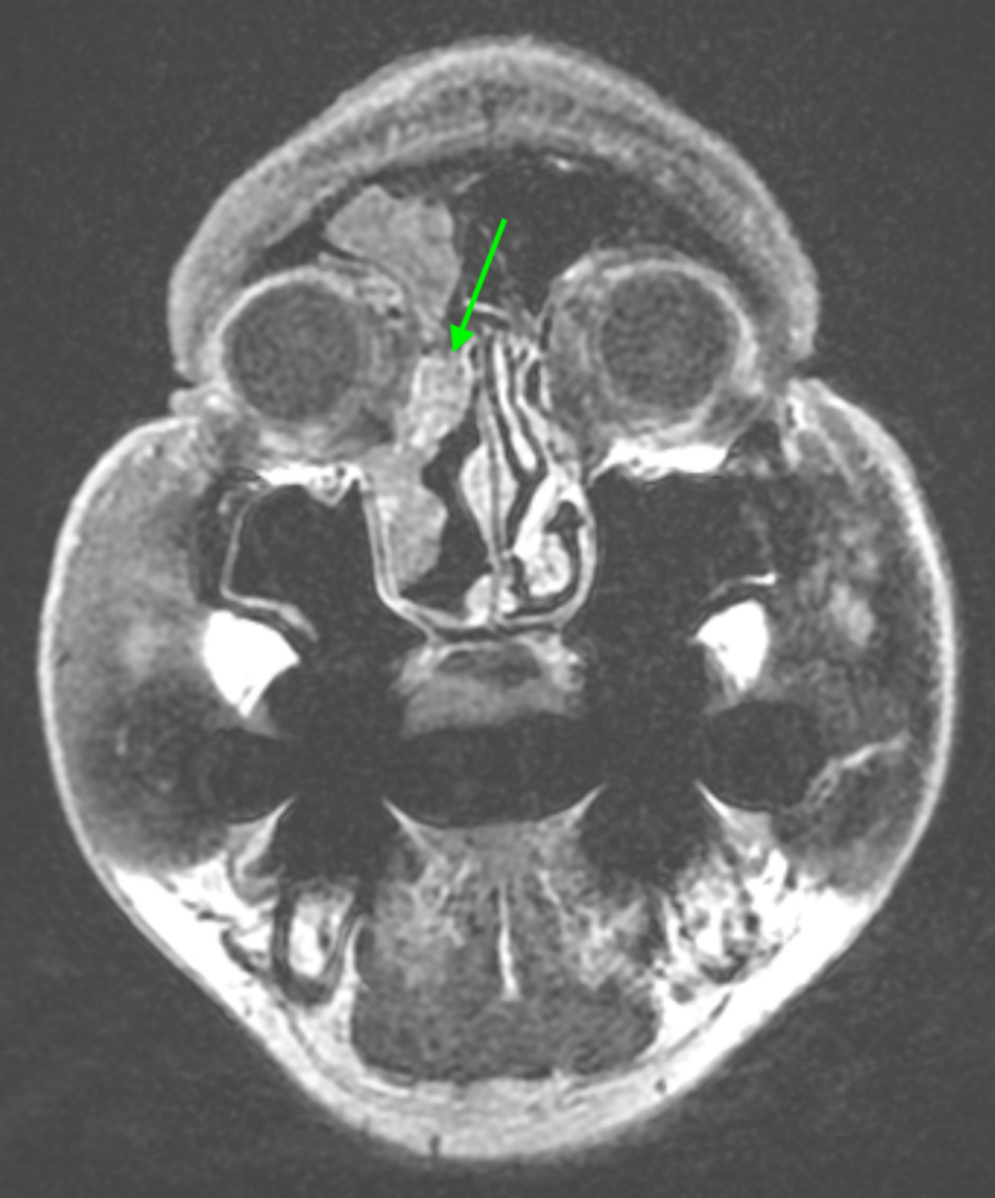
MRI showing recurrent tumor extending from the frontoethmoidal recess, along the lateral nasal wall.

Magnetic resonance imaging (MRI) showed a mass extending from the frontoethmoidal recess, along the lateral nasal wall into the nasal cavity (Figure 6).

**Figure 7:**
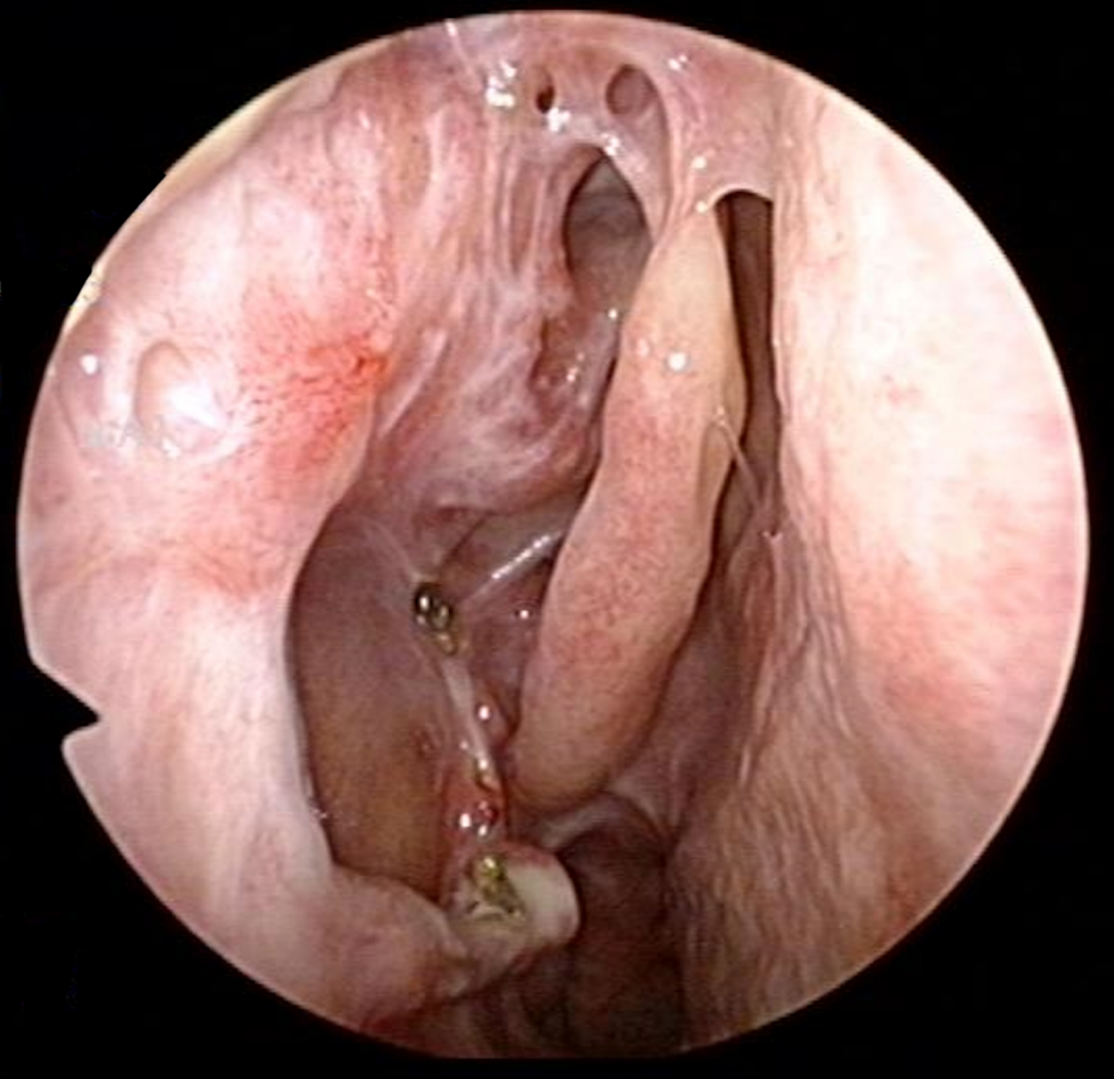
Nasoendoscopy image of right lateral nasal wall five years post-operatively, showing no evidence of tumor recurrence.

Intra-operatively, the tumor was noted to extend from the lacrimal fossa along the lateral nasal wall to the inferior meatus, and had infiltrated the remnant inferior turbinate inferiorly and frontal recess superiorly. Revision surgery was performed via the previous lateral rhinotomy incision, combined with endoscopic transnasal resection. Remaining superomedial aspects of the maxillary bone weret removed to expose the tumor around the lacrimal fossa completely and resection was performed. Intra-operative frozen section and final histology margins were clear, and the patient remains recurrence-free to date, five years post-operatively (Figure 7).

## Discussion

Inverted papillomas arising primarily from the lacrimal system are rare and have only been reported in isolated case reports. To date, there are fewer than 10 cases reported in the literature [1]. Inverted papillomas most commonly originate from the lateral nasal wall near the middle meatus, with subsequent contiguous spread to the maxillary and ethmoid sinuses [2,3]. These neoplasms are of particular concern to the clinician for several reasons. Despite being histopathologically benign, these tumors are known to be locally aggressive with a tendency to recur and have a malignant transformation rate, usually into squamous cell carcinoma, of approximately 5% [2,3]. Inverted papillomas arise from transitional epithelium within the nasolacrimal system, which has been postulated to result from metaplasia of respiratory epithelium secondary to chronic inflammation [4].

The most common presenting symptom is a medial canthal mass, followed by unilateral epiphora resulting from obstruction of the nasolacrimal drainage system [1]. Bloody epiphora, sanguineous refluxate during syringing and the presence of a firm, non-fluctuant swelling in the region of the medial canthus should alert the clinician to the possibility of a neoplastic lesion [5,6]. In the initial stages, inverted papillomas of the nasolacrimal system may present in a manner that is indistinguishable from primary acquired nasolacrimal duct obstruction or chronic dacryocystitis [6]. Invasion of the orbit is possible in advanced tumours, although this is a rare occurrence with only two cases reported in the literature [1].

Patients with neoplastic lesions of the nasolacrimal system often first present to the ophthalmologist because their presenting symptoms tend to be ophthalmological in nature. If there is a suspicion of nasolacrimal neoplasm, we recommend a referral to an otorhinolaryngologist for nasoendoscopy to identify possible extension of the mass lesion into the nasal cavity.

Cross-sectional imaging in the form of CT or MRI should be considered in all cases of suspected neoplasms of the nasolacrimal system, so as to establish the epicenter of the lesion and extent of involvement of surrounding structures [6]. This aids in planning for tissue biopsy and subsequent surgical resection. In cases where there is failure to suspect or absence of features suggestive of neoplasm pre-operatively and a dacryocystorhinostomy is performed for presumed primary acquired nasolacrimal duct obstruction or chronic dacryocystitis, the clinician should have a high index of suspicion and low threshold to biopsy if the lacrimal sac mucosa appears abnormal. In our case, despite the initial working diagnosis of a benign lacrimal mucocele, the lacrimal sac mucosa was biopsied because it appeared abnormally oedematous and papillomatous. Biopsy of the lacrimal sac mucosa is otherwise not recommended in routine dacryocystorhinostomy as it confers no added benefit [7,8].

The principles of management of inverted papilloma of the nasolacrimal system are similar to that of inverted papilloma arising within the nasal cavity or paranasal sinuses. Complete eradication of disease via wide surgical excision with clear margins is the mainstay of treatment [2,3]. Intra-operative confirmation of clear margins via frozen section is the standard of care in our institution.

Choosing the appropriate surgical approach is important as it determines visualization and access, which are critical in ensuring that the tumor is completely extirpated. The combined open-endoscopic approach has proven to be effective for resection of extensive benign neoplasms of the nasolacrimal system [9-11]. This approach provides access to both the most superior and inferior ends of the nasolacrimal apparatus, ensuring adequacy of resection while preserving much of the normal tissue architecture, unlike the more radical open approaches.

Following definitive surgery, close post-operative surveillance is necessary to identify any tumor recurrence early. Surveillance can be performed either via direct nasoendoscopic visualization or via CT or MRI scans if the primary tumor site cannot be adequately visualized on clinical examination. It has been shown that majority of recurrences for inverted papillomas occur within the first two years of definitive surgery [12]. However, late recurrences can also occur, and hence the need for long-term follow-up of these patients. A recommended surveillance schedule proposed by Suh et al. for sinonasal inverted papilloma categorizes patients into low- versus high-risk groups based on human papilloma virus positivity, Krouse or Cannady stage and any histologically aggressive features, in order to determine the frequency of follow-up. In general, at least three-monthly surveillance within the first post-operative year is recommended. Subsequently, regular interval follow-up every three to six months is continued up to five years post-operatively. Beyond five years, yearly review is deemed sufficient, not dissimilar to the surveillance schedule used in head and neck squamous cell carcinomas [13].

## Conclusions

The nasolacrimal system is an unusual and uncommon location for inverted papillomas to originate from. Meticulous history taking, physical examination and vigilant intra-operative observation are essential in diagnosis. CT or MRI is helpful in determining the extent of disease to aid in operative planning. The mainstay of treatment is complete surgical excision with clear margins, which is crucial to prevent tumor recurrence. Choosing the appropriate surgical approach is important and the combined open-endoscopic approach is one method that can maximize tumor visualization while minimizing surgical morbidity. Finally, long-term surveillance of these patients is recommended as tumor recurrence can occur even after five years.

## References

[1] Hardy AW, Dwivedi RC, Masterson L (2015). Inverted papilloma of lacrimal sac invading into the orbit: case report and review of literature. J Cancer Res Ther.

[2] Mendenhall WM, Hinerman RW, Malyapa RS (2007). Inverted papilloma of the nasal cavity and paranasal sinuses. Am J Clin Oncol.

[3] Wood JW, Casiano RR (2012). Inverted papillomas and benign nonneoplastic lesions of the nasal cavity. Am J Rhinol Allergy.

[4] Heathcote GJ (2012). Transitional neoplasms of the nasolacrimal system: a review of the histopathology and histogenesis. Saudi J Ophthamol.

[5] Heindl LM, Jünemann AG, Kruse FE, Holbach LM (2010). Tumors of the lacrimal drainage system. Orbit.

[6] Krishna Y, Coupland SE (2017). Lacrimal sac tumors: a review. Asia Pac J Ophthalmol (Phila).

[7] Bernardini FP, Moin M, Kersten RC, Reeves D, Kulwin DR (2002). Routine histopathologic evaluation of the lacrimal sac during dacryocystorhinostomy: how useful is it?. Ophthalmology.

[8] Merkonidis C, Brewis C, Yung M, Nussbaumer M (2005). Is routine biopsy of the lacrimal sac wall indicated at dacryocystorhinostomy? A prospective study and literature review. Br J Ophthalmol.

[9] Sullivan TJ, Valenzuela AA, Selva D, McNab AA (2006). Combined external-endonasal approach for complete excision of the lacrimal drainage apparatus. Ophthalmic Plast Reconstr Surg.

[10] Valenzuela AA, McNab AA, Selva D, O'Donnell BA, Whitehead KJ, Sullivan TJ. (2006). Clinical features and management of tumors affecting the lacrimal drainage apparatus. Ophthalmic Plast Reconstr Surg.

[11] Karkos PD, Fyrmpas G, Carrie SC, Swift AC. (2006). Endoscopic versus open surgical interventions for inverted nasal papilloma: a systematic review. Clin Otolaryngol.

[12] Mirza S, Bradley PJ, Acharya A, Stacey M, Jones NS (2007). Sinonasal inverted papillomas: recurrence, and synchronous and metachronous malignancy. J Laryngol Otol.

[13] Suh JD, Chiu AG (2014). What are the surveillance recommendations following resection of sinonasal inverted papilloma?. Laryngoscope.

